# Incorporation of MPCM on cotton fabric for potential application in hospital bed sheet

**DOI:** 10.1016/j.heliyon.2023.e16412

**Published:** 2023-05-22

**Authors:** Md Abdus Shahid, Champa Saha, Md Sumon Miah, Md Tanvir Hossain

**Affiliations:** a Department of Textile Engineering, Dhaka University of Engineering & Technology (DUET), Gazipur, Bangladesh; b Department of Apparel Manufacturing Technology, BGMEA University of Fashion & Technology (BUFT), Dhaka, Bangladesh; c Department of Textile Engineering, Bangladesh University of Business & Technology (BUBT), Dhaka, 1216, Bangladesh

**Keywords:** MPCM, Cotton fabric, Bed sheet, Screen printing

## Abstract

Over the last few decades, phase change materials (PCM) have attracted a great deal of interest in medical textiles due to its superior thermoregulation system, simple application, and so on. Patients, however, confined to bed in a medical facility face the serious risk of developing bed sores, which is not mitigated by the use of a standard bed sheet. Numerous articles and patents have been studied related to development of thermal bed sheets using PCM applied by various techniques; however, no such initiates was found to prepare and characterize of hospital bed sheets using microencapsulated phase change material (MPCM) through screen printing method. Thus, this study aims to develop a hospital bed sheet constructed from cotton fabric incorporated with MPCM. To accomplish this, MPCM was mixed into the printing paste that had been applied on the fabric by screen printing method, and then dried at room temperature. Thermal behavior, thermal transition, and thermal conductivity of the developed samples had been investigated. Moisture management properties, mechanical properties, and bonding behavior of the samples were also examined. Scanning electron microscope (SEM) was used to analyze the sample's morphology, and a differential scanning calorimeter (DSC) was used to determine how polymeric materials behaved when heated. Thermogravimetric analysis (TGA) demonstrated that the MPCM incorporated sample lost weight slowly, while the DSC test confirmed that melting began at 20 °C and ended at 30 °C. Furthermore, fabricated sample had higher heat conductivity (0.1760822 w.m^−1^ k^−1^). Overall, the results revealed a great potential for using the developed samples as hospital bed sheets to prevent patients from developing bed sores.

## 1 Introduction

PCM is using various branch of textile including medical textiles due to their distinctive capacity of soaking and emitting heat energy without altering the temperature over the years [[1], [2], [3]]. Besides textiles, PCM has been extensively used for various purposes such as food processing and space industries, sportswear, medical application, shoes and accessories, air conditioning of buildings and so on [[4], [5], [6]]. PCM is incorporating textile fabrics and apparels by coating or encapsulation techniques to manufacture smart textiles [[7], [8], [9]]. Cotton sheets are hypoallergenic and a good choice for sensitive skin, and PCM could reduce the temperature and humidity of its microenvironment and effectively alleviate the heat-stress phenomenon of the human body in hot temperature and high humidity environment, as well as improve the thermal comfort of bed sheets [7,10,11]. MPCM incorporated fabric can be used as a bedding material in the intensive care units (ICU) for thermal comfort, and researchers have tried using PCM with different fabric types via bath dipping technique [[12], [13], [14]]. PCM can be incorporated into fabric either through an oven curing process or by melting together synthetic polymers. Through melt compounding, modified plaques can be processed into monofilament and multifilament [15,16]. However, previous studies regarding the application of PCM on textiles have demonstrated that the durability, washing and rubbing fastness and performance of coated woven textiles are better than the melt compounded filament-based textiles [17]. In a search for alternative methods of applying PCM on bed sheet fabric, printing, coating or laminating techniques are gaining popularity due to their excellent thermal conversion efficiency, low price and affordability of the equipment [18]. Adding PCM with printing ingredients can be an ideal choice for applying it on the bed sheet. There has not been much study to use such novel approaches to incorporate the MPCM into the fabric by printing technique. Many researchers have analyzed phase change materials. Scacchetti et al. investigated multifunctional cotton fabrics using TiO_2_ and PCM, which is one of the most important studies because one or two-step finishing processes were investigated, and the results showed that the presence of PCM increased self-cleaning activity, thermoregulation properties, and antimicrobial properties [19]. Microcapsules were introduced to polyester knit fabrics via a typical pad-dry-cure technique to generate thermo-regulating textile materials, according to Younsook Shin et al. reported the washing durability test was assessed for practical application [20]. Shahid et al. created microencapsulated phase change materials using jute fabric for thermal and breathability control using a pad dry curing process, where water repellency qualities improved in the developed sample while air permeability values decreased [10]. In addition, flame-retardant PCM for firefighting protective clothing were reported by Su and his colleagues [21]. Champa et al. synthesized and characterized woven and knit fabrics that were treated with micro capsulated PCM at various concentrations through printing followed by a curing process to fix it and to create thermo regulation properties [22]. Sandra and coworkers used multiple knitted fabric structures with cushioning to develop microencapsulated phase change materials for firefighting clothing. Using microcapsules to finish the surface was examined for durability [22]. Iqbal et al. formed Glauber's salt nano-encapsulated with a PMMA shell and applied it to cotton to regulate temperature. On cotton, they compared its durability to that of commercial PCM and discovered that Glauber's salt nano-capsules' smaller size allowed for a stronger attachment to the fabric, even after washing [23]. Doba Kadem et al. studied the effects of processing cotton twill weave clothing with PCM in various concentrations using a dipping and curing method. According to DSC analysis, the treated clothing had better post-wash heat storage retention, with 80% of its capacity still present after five washings [24]. As based of our knowledge, MPCM based hospital bed sheet preparation and characterization using screen printing method has not been reported yet. Therefore, the purpose of conducting this study is to enhance the ability of bed sheets to regulate temperature and humidity for patients who are bedridden.

## 2 Materials and methods

### 2.1 Materials

A bed sheet comprised of 100% cotton fabric with twill (2/1) weave construction, EPI 104, PPI 54, fabric width 58-inches, warp count 19 Ne, weft count 13 Ne, and GSM 410 was used. The bed sheet was collected from the Nippon Garments, Tongi, Bangladesh. Paraffin wax, binder, titanium, and pigment gum were compiled from Alauddin Textile Mills Ltd, Tangail, Bangladesh. Poly-methyl methacrylate was sourced from Jonaki scientific store, Hatkhola road, Dhaka. All of the above chemicals were used without further purification during the experiments.

### 2.2 Fabrication process of thermo-regulating cotton fabric

MPCM was produced for textile applications by dispersing paraffin wax after it had been melted in a carrier material like poly-methyl methacrylate. The resulting mixture is then placed through an extrusion procedure to make microcapsules. Core material of MPCM was paraffin wax (70%) and the shell material was poly-methyl methacrylate (30%).

**Fig. 1:**
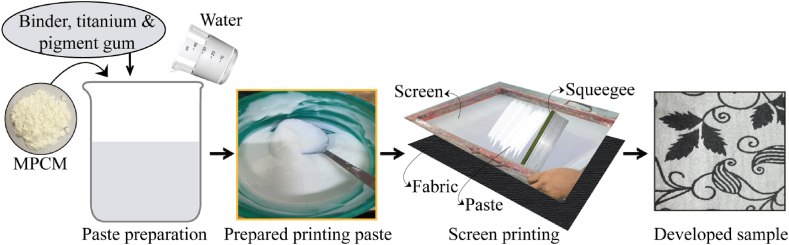
Schematic diagram to develop MPCM treated sample.

80 g of binder, 100 g of titanium, 8 g of pigment gum, and 70 g of MPCM were mixed in a container to prepare the printing paste. Then, 742 g of water was added to its while it was being continuously stirred for 30 min. The fabric was placed on a flat printing table and the screen was placed on it. The paste was taken on the screen. A rubber squeegee was used on the screen to apply the printing paste uniformly to the fabric. To facilitate the fixation of MPCM with the fabric, the printed sample was dried at room temperature. A thermo-regulating cotton fabric was developed according to schematic diagram of Fig. 1.

### 2.3 Characterizations

The surface morphologies of the cotton fabrics before and after treatment with PCM microcapsule were observed by scanning electron microscope (SU1510, Hitachi, Japan), and a portable microscope (digital microscope, juision, China) at a magnification of 40× to1000×.

In compliance with ASTM standards, thermo-gravimetric tests were performed using an ELTRA thermoset thermo-gravimetric analyzer (SDT 650, Discovery, USA). An alumina crucible with a minimum 400 mg sample was used for testing. The procedure involved heating at a rate of 5 °C/min from ambient temperature to 500 °C in an inert atmosphere with nitrogen gas.

DSC 60, Shimadzu, Japan was used to examine the phase transition temperature of MPCM. It heated the material from 0 °C to 100 °C at a rate of 10 °C per minute.

The chemical groups were recognized and matched using FTIR. Samples were exposed directly to an IR beam while being observed by an attenuated total reference of FTIR. For this, an infrared spectrometer with Fourier transforms called the IR prestige 21 (Shimadzu Corporation, Japan) was employed.

The materials of which the thermal conductivity to be determined should be cut in to test specimen of 20.6 mm diameter. The modified Lees' disk device and ASTM D- 7340 were used to measure thermal conductivity of the sample using Equation (i).

(i) $$Thermal\;Conductivity\;\left(K\right)=\frac{ed}{{2\pi r}^{2\;\left(T_{B}-T_{A}\right)}}\;\left(2a_{A}T_{A}+a_{s}\frac{T_{a}+T_{B}}{2}\right)$$

where,

e = Amount of heat energy transferred from heat to cold end

d = Thickness of sample (m)

L = Width of material in machine = 12.7 × 10^−3^ m.

I = Width of heater in machine = 4.8 × 10^−3^ m

r = radius of circular copper disc = 2.06 × 10^−2^ m.

T_A_ = Temperature of disc A (°C)

T_B_ = Temperature of disc B (°C)

Tc = Temperature of disc C (°C)

a_A_ = Surface area of disc A = a_A_ = nr2 + 2nrl = 29.755 × 10^−4^ m^2^

a_B_= Surface area of disc B = a_B_ = 2nrl = 16.430 × 10^−4^ m^2^

ac = Surface area of disc C = a_C_ = nr2 + 2nrl = 29.755 × 10^−4^ m^2^

a_S_= Surface area of sample = 2nrd m2

a_H_= Surface area of heater = 2nrl = 6.21x 1O^−4^ m^2^

V = One of the potential differences across element in volt = 6.4 V.

I= The current flowing in ampere = 0.35 A.

W = VI = 2.24 J/Sec = 2.24 W.

AATCC 195–2009 states that a moisture management tester (model: M290, SDL Atlas, UK) was used to evaluate sample's capacity to control moisture.

A universal tensile testing device (M250-3CT, Testometric, UK) with a precise load cell capacity range of 112.4 lbs. was used to assess the tensile properties of the samples according to ASTM D 5034 2001 method with testing speed of 11.8 inches per minute.

The surface roughness property of fabrics was measured with fabric touch tester (FTT) (model: M293, SDL Atlas).

## 3 Results and discussion

The surface morphology and microstructure of the MPCM were examined using SEM, while TGA was used to determine the thermal stability and decomposition of the developed sample, and DSC was used to measure the thermal properties and phase transitions. Besides, a moisture management test was performed in order to gain insight into the moisture and absorption characteristics of the material. Thermal conductivity measurements were also used to evaluate the material's capacity to conduct heat. In addition to these tests, tensile strength and surface roughness measurements of the material's strength were combined to evaluate the material's resilience and durability in order to better understand its performance. Comparing the results of these tests between the sample with MPCM and without MPCM, a comprehensive understanding of the material's overall performance was obtained, allowing for a more thorough evaluation of its suitability for various applications.

### 3.1 Surface morphology of MPCM

**Fig. 2:**
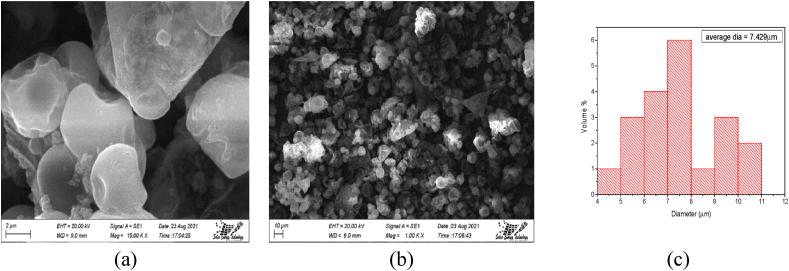
Image of MPCM at (a) 15.00 k; (b) 1.00 k magnification and its (c) diameter distribution with respective frequency.

The PCM microcapsule powder morphologies are shown in Fig. 2. Fig. 2(a) displays the MPCM picture at a magnification of 15.00 k, Fig. 2(b) displays the MPCM images at a magnification of 1.00 k, and Fig. 1(c) displays the distribution of measuring diameters with frequency. PCM microcapsule powder had a smooth surface morphology, consistent forms, and was spherical. The PCM microcapsules' spherical shape, which had higher surface tension and was simple to distribute, will be helpful for subsequent processing. Furthermore, no damaged capsules were found, indicating a high degree of encapsulation.

#### 3.1.1 Size distribution of MPCM

A laser particle size analyzer was used to measure the size distributions of microcapsule powder, and also displays the frequency-dependent distribution of MPCM powder diameter. The diameters of the microcapsule powder ranged from 4 μm to 9 μm. The average diameter of the 20 samples of powder molecules that were examined in various areas of the image was 7.429 μm. The powder's minimum and maximum diameters were 4.058 μm and 10.915 μm, respectively.

#### 3.1.2 SEM analysis of the MPCM applied fabric

**Fig. 3:**
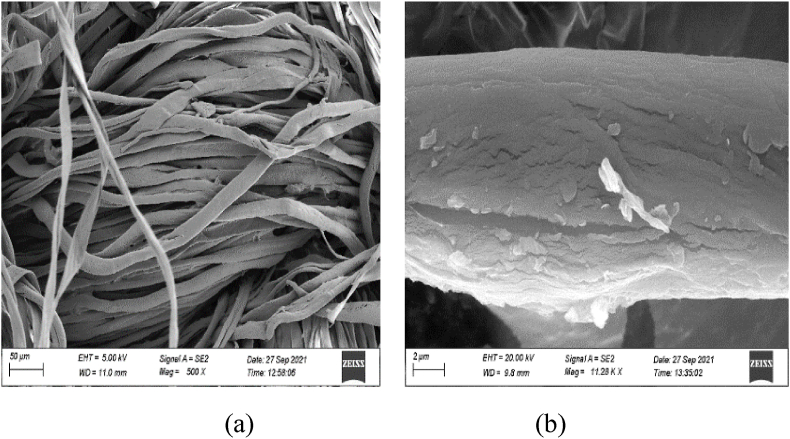
SEM image of fabric surface (a) without MPCM and (b) with MPCM.

SEM pictures of the samples with and without MPCM cotton twill weave fabric are shown in Fig. 3. Higher retention on treated fabrics may be achieved by choosing the right binder and application technique. Additionally, gentle washing conditions might be beneficial for improved upkeep of materials treated with microcapsules [12].

The surface of the cotton fabric with 50 μm magnification is shown in Fig. 3(a) while Fig. 3(b) represents the surface morphology of a fiber with 2 μm magnifications. Each photo indicates a well incorporation of MPCM on the cotton fabric surface.

### 3.2 TGA

**Fig. 4:**
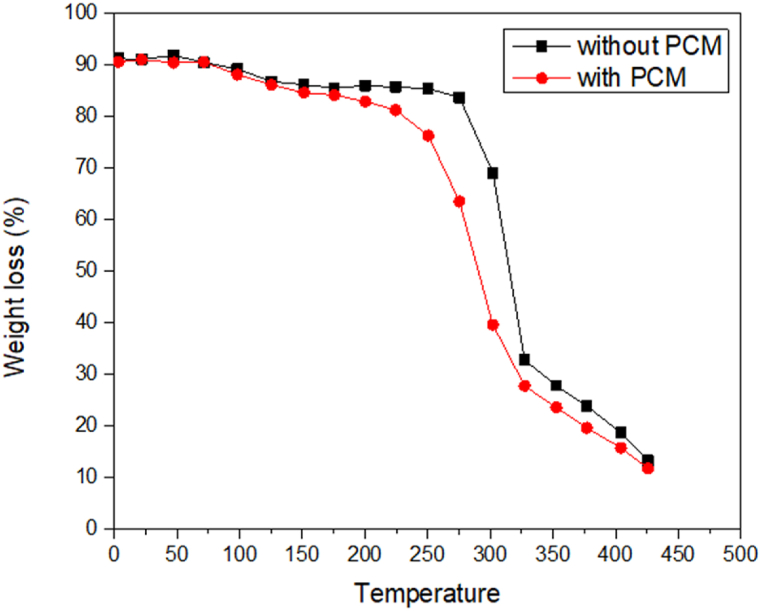
Effect of temperature on weight loss of the experimented sample.

Fig. 4 displays the thermal behavior of fabrics with and without MPCM inclusion in the shape of a curve. Weight decreased dramatically with rising temperature in the sample of without MPCM. The MPCM incorporated sample, on the other hand, showed substantial weight retention and a slowing down of weight loss. This suggests that the heat was directly absorbed by the fiber in the sample without MPCM, and weight retention was lower. The samples' moisture escapes as a result of absorbing the heat, and the heat then leads to the loss of fiber weight. The developed sample, on the other hand, showed a less significant change in the weight of fiber. Due to the MPCM printed layer is damaged by heat. The moisture is then helped to leave the fibers with the aid of heat, which results in a loss of fiber weight. Consequently, after being exposed to heat, compared to untreated fabric, the produced fabric would be more thermally stable. In contrast, the weight retention percentage of developed sample compared to without MPCM fabric is remarkable in the case of TGA. Without MPCM sample's consistent weight loss suggests a weak heat resistance, which is why the weight of the sample decreases proportionately to the increase in temperature [25].

### 3.3 DSC analysis

**Fig. 5:**
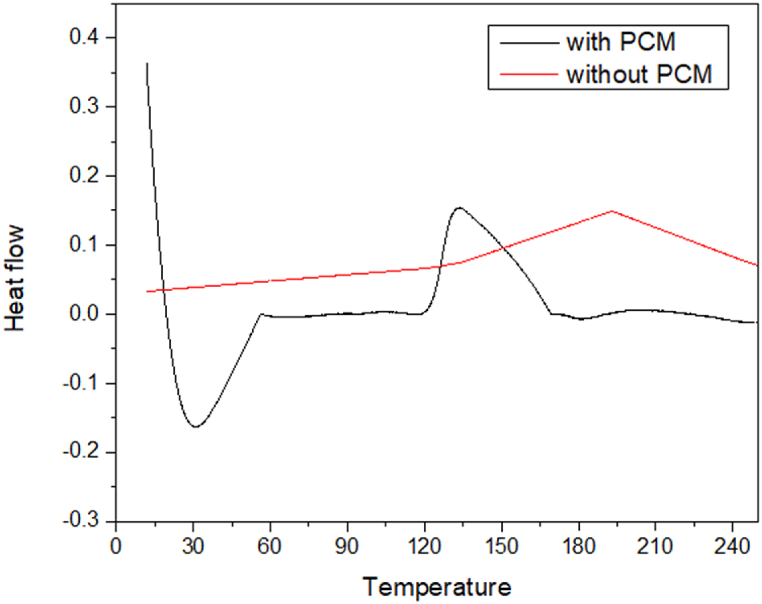
DSC curve of fabrics with and without MPCM.

Fig. 5 provides the DSC thermograms of fabrics with and without MPCM addition. The baseline is corrected for the peak due to the melting. The MPCM begins changing phase from solid as soon as the temperature approaches its melting point of 30 °C is indicated by the melting peak while there is no peak for this phase transition in the fabric without MPCM. Actually, in reality, the MPCM's [26,27] phase transition only happens within a certain temperature range. It is supported by the phase change peak area. When the melting curve is examined more closely, it can be seen that the melting starts at 20 °C and concludes at 30 °C. The manufactured microcapsules crystallized at a temperature of 133.4 °C. As a result, it has been found that cotton twill weave fabric incorporated with MPCM has better thermoregulation. This is due to the MPCM's ability to absorb heat, which stops the temperature from rising or falling. However, the treated samples demonstrate that there was a nearly linear or negligible weight reduction. This could be attributable to MPCM, which helps to prevent overheating by absorbing heat flow up to a particular point, or 30 °C. It signifies that the heat flow from an outside source for the fiber is being blocked by MPCM microcapsules; hence the fiber cannot be heated even though the heat flow is present. Although the figure for MPCM incorporated sample is relatively low, weight loss happens as the temperature is above the melting point of MPCM microcapsules. The latent heat and heat conductivity of the MPCM material was 1655 J/g and 0.21 w/m^o^C respectively.

### 3.4 FTIR analysis

**Fig. 6:**
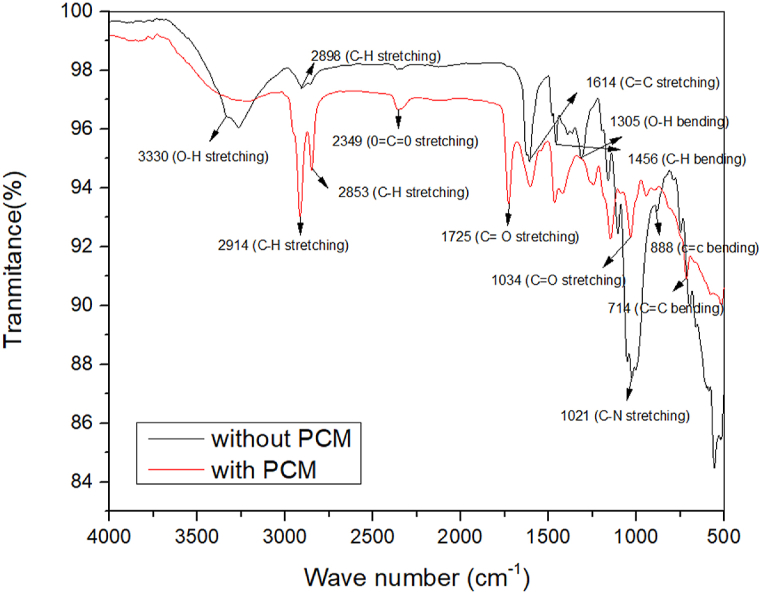
FTIR spectra of fabrics with and without MPCM.

Fig. 6 shows how infrared spectroscopy confirmed the cellulose polymer's functional group was present. Using FTIR spectroscopy, the chemical difference between the functional groups of ordinary cotton fabric and cotton fabric with MPCM was identified. The spectrum of cotton exhibits the characteristic bands of cellulose, lignin, and hemicellulose. The hydroxyl (OH) groups of cellulose, lignin, and water are what give off the prominent peak at 3330 cm^−1^ its characteristic appearance [28]. The band at 1614 cm^−1^ may be related to the presence of water in the fibers, and the peak at 2896 cm^−1^ is typical of the stretching vibration of C–H seen in cellulose and hemicellulose [29].

The absorption band at 1428 cm^−1^ is associated with the CH_2_ symmetric bending of the cellulose. The absorption bands at 1305 cm^−1^ are relative to bending vibrations of the C–O group, respectively, of the aromatic rings in cellulose polysaccharides. The (CO) and (OH) stretching vibrations of the polysaccharide in cellulose are responsible for the strong peak vibrations seen at 1021 cm^−1^. The signal at 888 cm^−1^ shows that monosaccharide to β-glyosidic connections are present [30]. Additionally, the paraffin chains of PCM contributed to the observation of an increase in the intensity of the absorption bands associated with the stretching vibration of CH at the ranges 2914 cm^−1^ and 2854 cm^−1^ [31]. The ester group (O=C=O) stretching vibration has been attributed to the 2349 cm^−1^ area of the spectrum [32,33]. The stretching vibration of the ester bond (C–O) can be used to explain the broad peak spanning from 1260 to 1000 cm^−1^. The linear saturated aliphatic structure of the paraffin wax is supported by the CH_2_ absorption band at 714 cm^−1^ [19].

### 3.5 Thermal conductivity

Since, thermal conductivity has a significant influence on the comfort properties of the fabric especially for the bed sheet for patients, it was measured by the modified Lees's disk device and it is shown in Table 1. This metric, which mostly depends on the structure, shows how well solid materials transport heat [34]. After analyzing the results, it was found that the samples treated with MPCM had more thermal conductivity. The thermal conductivity appeared 0.1680864 w.m^−1^ k^−1^ for the fabric without MPCM while 0.1760822 w.m^−1^ k^−1^ appeared for fabric with MPCM. Since the thermal conductivity is slightly increased but underlined in the comfort range. However, it will not destruct the comfort properties of the bed sheet. Since, available thermal conductivity reported in Ref. [35] as 0.243 w.m^−1^ k^−1^ for cotton, 0.344 w.m^−1^ k^−1^ for flax fiber. It is well recognized that the usage of MPCM in textile fabrics provides users with thermal comfort. In addition, when comparing the side of treatment side, the thermal conductivity of the functionalized samples increased, according to the thermal conductivity equation of these data. Even while all of the samples had increased conductivity, the MPCM incorporated sample showed the biggest increase [36]. Moreover, due to their thickness, heat conductivity in this area reduces by 0.8%.

**Table 1:** Thermal conductivity of fabrics with and without MPCM.

Sample	Sample thickness (mm)	Thermal conductivity (w.m^−1^ k^−1)^
Fabric without MPCM	0.99	0.1680864
Fabric with MPCM	1.02	0.1760822

### 3.6 Moisture management properties

**Fig. 7:**
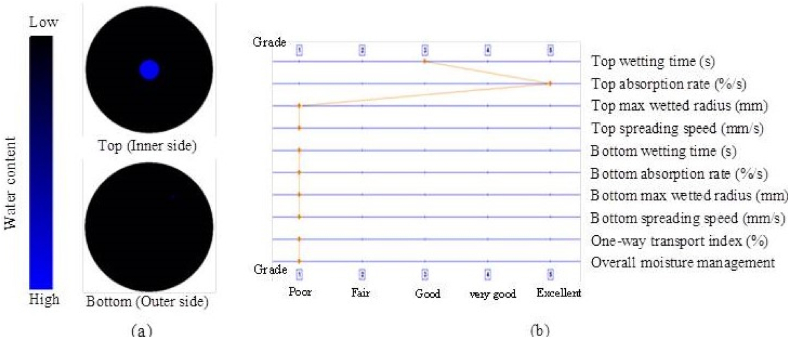
(a) Water placement diagram and (b) fingerprint of the fabric with MPCM.

**Fig. 8:**
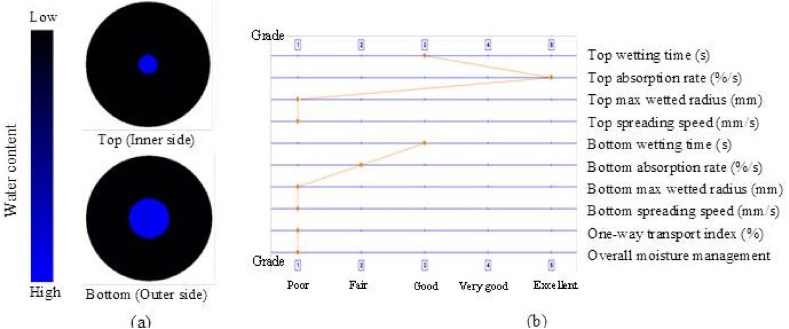
(a) Water placement diagram and (b) fingerprint of the fabric without MPCM.

The ability of the textile material to manage moisture is crucial. As a result, the qualities of moisture management were assessed for the water transfer from the outside environment to the inside of the cotton twill weave fabric, as illustrated in Fig. 7(a and b), Fig. 8(a and b) and Table 2 respectively.

**Table 2:** Moisture management properties of fabrics with and without MPCM.

	Wetting time (S)		Absorption rate (%/s)		Max Wetted radius (mm)		Spreading speed time (mm/s)		AOTI (%)	OMMC
	Top	bottom	Top	bottom	Top	bottom	Top	Bottom		
Without MPCM	18.8	120	328.5	0	5	0	0.26	0	−662.4	0
With MPCM	15.6	77.6	370.2	13.9	5	5	0.32	0.3	−486.6	0.04

*OMMC- overall moisture management capacity.

*AOTI- accumulated one-way transport index.

The produced sample's top and bottom surface wetting times were 18.8 s and 120 s compared to 15.6 s and 77.6 s for the without MPCM fabric. It indicates that when water contacts the MPCM incorporated sample's outer face, it takes a lot longer to spread than it does on the sample without MPCM. The material may not soak up water very quickly due to the nature of the generated sample. The sample without MPCM has a poorer top absorption rate of 370.2%/s compared to the improved sample's 328.5%/s. In contrast to the sample without MPCM, this showed bottom wetting and absorption of 5 mm and 13.9%/s. The sample with MPCM showed no evidence of either. The produced sample's total humidity operation was zero (0), compared to 0.04 for the PCM cotton twill weave fabric. Due to the hydrophilic nature of the treatment process, the generated sample's 0 humidity operation reflects a fabric that is largely water resistant [37]. In actuality, the tempera-styrene binder that is attached to the MPCM is the hydrophobic component on the fabric face, which negates the generated sample's ability to operate under humidity [38,39]. As a result, it was revealed that the developed sample possesses moisture management properties that were suitable for imparting comfort to the patients.

### 3.7 Tensile properties evaluation

**Fig. 9:**
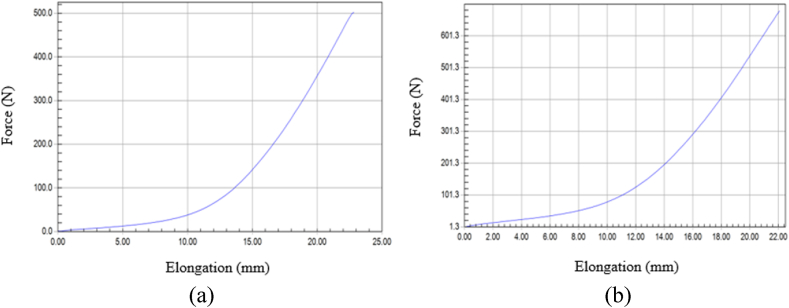
Tensile behavior of fabrics (a) with MPCM and (b) without MPCM.

This study's objective was to evaluate newly created structural cotton twill weave textiles' tensile property after being subjected to an abrasion load and to contrast them with and without MPCM coated fabrics. Their mechanical characteristics, particularly their tensile strength, are crucial. A tensile test was carried out and plotted on the force-extension curve as given in Fig. 9. To examine the pulling forces, it can withstand before breaking. According to Fig. 9(b), in fabrics without MPCM, breaking force is 502 N and the elongation value is 22.8 mm. According to Fig. 9(a), the maximum breaking force of the fabrics with MPCM was found 679 N whereas corresponding to the elongation value is 28.9 mm. From the results, it can be observed that there is a significant change in warp way tensile strength as compared to weft way tensile strength, which is sufficient to use as hospital bed sheet. An increase in the warp and weft way strength of the developed sample may be a result of extra bonding between the fibers provided by the poly-methyl methacrylate coating [40].

### 3.8 Surface roughness

**Fig. 10:**
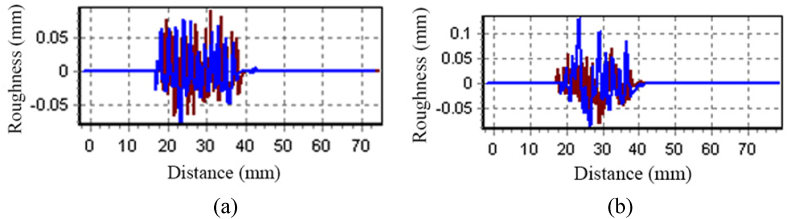
Surface roughness of fabric (a) without MPCM and (b) with MPCM.

There is a critical need for surface roughness testing for MPCM incorporated cotton fabrics used in hospital bed sheets since it can affect the fabric's thermal regulation performance as well as overall comfort for patients. There are a number of factors that can affect the distribution of PCM on the fabric's surface as well as its adhesion to it, which affects the amount of thermal energy that is stored and released on the fabric. It has been proven that a smooth surface with a proper distribution of MPCM is able to provide more effective thermoregulation, which ensures the patients' comfort during prolonged stays in hospital beds [41]. Surface roughness value of fabrics with and without MPCM is provided in Table 3. Surface roughness image of fabric without MPCM and with MPCM are given in Fig. 10(a) and Fig. 10(b) respectively.

**Table 3:** Surface roughness of fabrics with and without MPCM.

Axis	Without MPCM	With MPCM
X(gf)	0.078	0.000
Y(gf)	−0.029	0.000

As shown in Fig. 10 and Table 3, the printed materials form a layer which produces an even surface roughness. Due to the interlacing points of the fabric's warp and weft yarn, the printed material fills the fabric's uneven areas evenly. Warp and weft yarns create the fabric's crimpy surface structure. The fabricated sample with MPCM, however, covers the staple fiber end, resulting in a relatively even surface [42].Thus, it is expected that the developed sample would be suitable for the hospital bed sheet applications.

## 4 Conclusion

The study successfully developed a medicated bed sheet from cotton fabric with MPCM of 7.429 μm through screen printing, which was the primary focus of this research aimed at exploring the utilization of MPCM in cotton fabric using a simple printing technique. The properties of the developed samples were analyzed, including TGA, DSC, thermal, moisture management, mechanical, and bonding behavior. The TGA results showed that the weight of the sample without MPCM declined dramatically with rising temperature, whereas the weight of the sample with MPCM retained significant weight and slowed weight loss. DSC analysis established the peak at 30 °C for the phase change of the MPCMs. The FTIR spectra of the sample without MPCM confirmed the presence of an only a functional group of the cellulose polymer. Furthermore, the increase in the intensity of the absorption bands related to stretching vibration of CH at the ranges 2914 cm^−1^ and 2853 cm^−1^ was observing due to the contribution of paraffin chains of PCM. The CH2 absorption band at 714 cm^−1^ confirms the linear saturated aliphatic structure of the paraffin wax. The developed sample can absorb excess moisture from the surrounding, thus reducing the temperature and humidity within the fabric. Before incorporation of MPCM in the fabric, force and elongation was 502 N and 22.8 mm respectively and after incorporation of MPCM in the fabric, it was 679 N and 22.9 mm respectively. The developed sample with MPCM had greater heat conductivity (0.1760822 w.m^−1^ k^−1^) compared to the sample without MPCM. Surface roughness affects the fabric's thermal regulation performance as well as overall comfort for patients. The created sample, however, covers the staple fiber end, resulting in a relatively even surface. Finally, this study could lead to new possibilities for hospital beds that prevent bed sores for patients.

## Author contribution statement

Md Abdus Shahid: Conceived and design the experiments; Performed the experiments; Analyzed and interpreted the data; Contributed reagents, materials, analysis tools or data.

Champa Saha: Performed the experiments; Analyzed and interpreted the data; Contributed reagents, materials, analysis tools or data; Wrote the paper.

Md Sumon Miah, Tanvir Hossain: Analyzed and interpreted the data; Contributed reagents, materials, analysis tools or data, Wrote the paper.

## Data availability statement

Data included in article/supplementary material/referenced in article.

## Additional information

No additional information is available for this paper.

## Declaration of competing interest

The authors declare that they have no known competing financial interests or personal relationships that could have appeared to influence the work reported in this paper.
